# There Is Light and There Is Darkness: On the Temporal Dynamics of Cohesion, Coordination, and Performance in Business Teams

**DOI:** 10.3389/fpsyg.2019.00847

**Published:** 2019-04-24

**Authors:** Pedro Marques-Quinteiro, Ramón Rico, Ana M. Passos, Luís Curral

**Affiliations:** ^1^William James Center for Research, ISPA – Instituto Universitário, Lisbon, Portugal; ^2^Business School, University of Western Australia, Perth, WA, Australia; ^3^Business Research Unit, ISCTE – Instituto Universitário de Lisboa, Lisbon, Portugal; ^4^CICPSI, Faculdade de Psicologia, Universidade de Lisboa, Lisbon, Portugal

**Keywords:** team coordination, team cohesion, complex adaptive systems, team performance, latent growth curve models

## Abstract

This study examines teams as complex adaptive systems (tCAS) and uses latent growth curve modeling to test team cohesion as an initial condition conducive to team performance over time and the mediational effect of team coordination on this relationship. After analyzing 158 teams enrolled in a business game simulation over five consecutive weeks, we found that change in team coordination was best described by a continuous linear change model, while change in team performance was best described by a continuous nonlinear change model; and the mediation latent growth curve model revealed a negative indirect effect of team cohesion on the level of change in team performance over time, through the level of change in team coordination. This study contributes to the science of teams by combining the notions of initial conditions with co-evolving team dynamics, hence creating a more refined temporal approach to understanding team functioning.

## Introduction

Team cohesion is an emergent affective state that is at the heart of teamwork dynamics (Kozlowski and Chao, 2012; Maynard et al., 2015). It is a multidimensional construct that includes a task, a social, and a group pride dimension. Accordingly, team cohesion is defined as the tendency for a team to stick together and remain united in its pursuit of instrumental objectives and the satisfaction of members’ affective needs (Carron and Brawley, 2000). Team cohesion is especially important for the performance of business teams (e.g., Menges and Kilduff, 2015). Indeed, since the early 50s (e.g., Festinger, 1950) teamwork literature has dedicated great attention to the relationship between team cohesion and team performance in organizational settings with cross-sectional, meta-analytical, and longitudinal studies suggesting a positive relationship between the two constructs (e.g., Zaccaro et al., 1995; Beal et al., 2003; Mathieu et al., 2015).

Thanks to the accumulating body of research we now know more about the dynamic nature of the cohesion–performance relationship. For instance, we know that the relationship between team cohesion and team performance (a) takes an inverted U-shaped distribution (Wise, 2014); (b) is stronger when performance is operationalized as behavior rather than an outcome; and (c) that efficiency measures are better for capturing this relationship (Beal et al., 2003). And yet, whereas the importance of team cohesion to team performance is unequivocal, the number of longitudinal studies trying to uncover the developmental dynamics between them is scarce (e.g., Kozlowski and Chao, 2012; Kozlowski, 2015; Mathieu et al., 2015). Studying how phenomena co-evolve over time is informative about how change in one construct can help explain change in another construct and how their influences reciprocate longitudinally (Selig and Preacher, 2009). Such an approach allows for a more in-depth examination of how teamwork dynamics happen; hence, clarifying what we know about how teams do their work (Ployhart and Vandenberg, 2010). Regarding the cohesion–performance relationship, this approach helps clarify previous debate on the dynamic nature of cohesion (Kozlowski and Chao, 2012). However, we also believe that more about the cohesion–performance relationship in management teams can be learned if framing cohesion as an initial condition for team performance trajectories over time is utilized.

In order to make progress in the temporal consideration of team cohesion dynamics and because their study cannot be dissociated from the study of time (e.g., Kozlowski and Chao, 2012; Mathieu et al., 2015); we build on the theory of teams as complex adaptive systems’ (tCAS) fundamental premise, that teamwork dynamics are sensitive to teams’ initial conditions at the beginning of any performance cycle (i.e., the period of time that starts with the commencement of a project or a mission and ends when the project or the mission is completed or fulfilled—Arrow et al., 2000; Marks et al., 2001). *Accordingly, this* study examines the team cohesion–team performance relationship from a new perspective: we test the general hypothesis that teams’ levels of cohesion, when a team begins a performance cycle, are an initial condition impacting team performance dynamics across the entire duration of the performance cycle. Furthermore, the theory of tCAS also suggests that team cohesion is an initial condition to the developmental dynamics of team performance and that this relationship should be driven by the developmental dynamics of team coordination (i.e., how team members manage their task interdependencies during goal-directed action—Rico et al., 2008).

Although the former affirmations are apparently logical and intuitively appealing, a black box remains in the teamwork literature since these affirmations remain neglected inside the team cohesion–team performance causal link. To redress this situation, in this study we contribute to extant literature by integrating longitudinal theory with the theory of tCAS and the episodic framework of team processes to disentangle the developmental dynamics of team cohesion, team coordination, and team performance (Arrow et al., 2000; Marks et al., 2001; Kozlowski and Chao, 2012; Mathieu et al., 2015). Through our research we will help address the question of why and how team cohesion is related with team performance over time. We examine which are the forms of change that team coordination and team performance take over time, and how such change relates to team cohesion as an initial condition, from the beginning to the end of a business management simulation competition.

### Theoretical Background

Complex adaptive systems (CASs) are central for dynamical systems (NDS) theory (Lewin, 1993). Under this theoretical framework, tCAS are regarded as “a set of independent agents acting in parallel to develop models of how things function in their setting, and to refine such models through learning and adaptation (…) CAS are open systems characterized by uncertainty about their evolution over time, due to the interaction of their components” (Ramos-Villagrasa et al., 2018, p. 136). According with Arrow et al. (2000), team dynamics are characterized by emergent interactions between local (i.e., team members characteristics), contextual (i.e., team processes and emergent states like coordination and cohesion), and global dynamics (i.e., contextual features such as task) as they unfold over time. These interactions drive teams toward self-organization, which is an optimum state of team functioning where teams become fully adapted to the task and/or the environment in which they are performing. Occasionally, either driven by internal or external triggers, the relative stability that exists in self-organized states is disrupted. Such discontinuities in team functioning are well documented in the work of authors such as Gersick’s (1991) punctuated equilibrium model, or Uitdewilligen et al. (2018), who found that team processes and team performance unfold over time through longer periods of stability, which alternate with shorter periods of instability where discontinuities occur.

Roe (2008) framework helps in integrating the aforementioned perspectives by suggesting that the dynamic relationship between constructs can be understood via paired combinations of three temporal features: the beginning of phenomena, which describes the initial value of any given variable (i.e., the onset/ intercept); the change in phenomena, which describes the form, direction, and intensity of development (i.e., the slope); and the duration in phenomena, which is the amount of time phenomenon persists, is observable, or behaves in a particular way (Roe, 2008). In this study, we focus on the beginning of phenomena addressing team cohesion as an initial condition; and on the dynamics of phenomena addressing the evolution of team performance *via* team coordination over time.

### Team Cohesion as an Initial Condition to Change in Team Performance Over Time

Team cohesion is considered of greatest importance for team performance over time. Team cohesion emerges in the early stages of the team life cycle, stabilizes quickly, and is expected to become a *sine qua non* condition to the integrity of teams (Festinger, 1950; Weiss and Cropanzano, 1996; Arrow et al., 2000). Cohesion is understood as a performance antecedent, and research findings have systematically shown a positive relationship between both constructs (e.g., Gully et al., 1995; Beal et al., 2003). However, few studies have examined this relationship from a longitudinal lens, despite the advantages that collecting data longitudinally entails clarifying the relational patterns between constructs that are hardly identifiable in data collected on a single occasion (Roe, 2008). In this regard, research by Mathieu et al. (2015) found meta-analytical support to the reciprocal influence between cohesion and performance over time in management teams. Mathieu et al. (2015) further extended this finding by conducting two empirical studies where they found that cohesion and performance were related positively, and reciprocally, over time. Their longitudinal model worked best when cohesion predicts performance over time, but not the other way around.

By framing team cohesion as an initial condition to team performance dynamics over time we are not ignoring the temporal nature of cohesion, nor its dynamic relationship with performance; but rather acknowledging the role that cohesion levels at early stages of a team performance cycle might have predicting how and why different teams show distinct performance trajectories over time. Building on Arrow et al. (2000) and Roe (2008), we theorize that team cohesion is an initial condition to teamwork dynamics over time. Our argument is also built over Hackman’s (2012) idea of team enabling conditions, which are regarded as the optimal set of team conditions (e.g., affectivity, knowledge) at the beginning of a project or a mission, that will set the stage for a team to be the most effective it could be. Consequently, and by combining the ideas of Roe (2008); Hackman (2012), and Arrow et al. (2000), we propose that high cohesion levels at the beginning of a performance cycle will be positively related with team performance dynamics across one complete performance cycle.

Team cohesion builds the teams’ structures that allow team members to engage in open communication, debate their ideas, and learn from each other (e.g., Festinger, 1950; Mathieu et al., 2015; Maynard et al., 2015). This means that, in cohesive teams, when teams start defining a plan or a strategy, team members will more confidently participate in its elaboration. The fact that teams have higher cohesion at the beginning of a task might also be helpful if it encounters some kind of obstacle early in the team performance cycle because more cohesive teams will be more likely to work together to overcome such an obstacle. Additionally, teams that begin a project or a mission with high cohesion levels have a strong sense of mission and are more willing to invest in helping the team to achieve its goals (Kozlowski and Chao, 2012).

In contrast, for teams with low cohesion at the beginning of a new performance cycle it is less likely that team members will feel motivated to fully invest their efforts in the achievement of the team’s goals, or that all team members will contribute to the definition of a team strategy (e.g., Zaccaro et al., 1995). Plus, the low cohesion levels at the beginning of a performance cycle might facilitate the emergence of conflict, which will impair team members’ collective capacity to work together and perform well over time (Kozlowski and Chao, 2012). Following these arguments, we propose that at the beginning of a performance cycle, cohesion will function as an initial condition that promotes positive performance trajectories over time. Thus, we hypothesize that:

*Hypothesis 1:* The level of team cohesion at the beginning of the team performance cycle is positively related with the level change in team performance over time.

Because the way teamwork dynamics develop over time can display different patterns (e.g., continuous vs. discontinuous; linear vs. nonlinear), it is first necessary to elaborate on the changing dynamics of team performance (e.g., Ployhart and Vandenberg, 2010; Navarro et al., 2015). Later in this section, we will do the same for team coordination.

The minimum entropy principle suggests that efficient performance in tCAS can only be achieved if systems develop a minimum number of alternative behavioral strategies that they can use to adapt to their environment (Arrow et al., 2000; Guastello et al., 2013). It is the existence of a minimum number of behavioral options that allows tCAS to be effective (Arrow et al., 2000). Interestingly, although high performance is often regarded as the most desirable outcome in the teamwork literature, the minimum entropy principle suggests that some variability in performance is what allows the system to thrive in the face of change and uncertainty (Ramos-Villagrasa et al., 2012; Guastello et al., 2013; Curral et al., 2016). It is as if living-social systems need to alternate between moments of high and low performance in order to secure systems’ sustainability in the long term. This idea finds support in an accumulating body of empirical evidence showing that the dynamics of change in team performance over time have chaotic properties in the sense that change in the level of team performance has sensitiveness to initial conditions and follows a *nonlinear trend* (e.g., Guastello, 2010; Ramos-Villagrasa, et al., 2012; Guastello et al., 2013; Curral et al., 2016; Ramos-Villagrasa et al., 2018).

The minimum entropy principle is also supported by the idea of healthy variability, a property of living systems where healthy functioning only exists if those systems show a minimum degree of entropy in their functioning over time (Navarro and Rueff-Lopes, 2015; Ramos-Villagrasa et al., 2018). In living and social systems, rather than linear, curvilinear, or random variability, healthy variability is characterized by nonlinear dynamics in the sense that the level of change in one particular variable follows a *slightly disorganized* pattern of ups and downs (i.e., organized chaos). As an example, Ramos-Villagrasa et al. (2012) found that team performance dynamics showing healthy variability were related with higher team performance in the long term. The outcomes of their research also showed that team performance dynamics characterized by linear and random variation (unhealthy variability) were related with poorer team performance in the long term.

In line with previous findings and taking the view of tCAS, we expect that team performance developmental dynamics over time will be in line with the minimum entropy system and the healthy variability principle, i.e., team performance over time will change nonlinearly. Hence, we hypothesize that:

*Hypothesis 2:* Team performance dynamics over time will display a nonlinear trajectory across the performance cycle.

### Team Cohesion as an Initial Condition to Change in Team Coordination Over Time

It is through team coordination that teams implement their strategy to achieve collective goals (e.g., Schmutz et al., 2015). Coordination happens when team members manage their multiple interdependencies. It regards the intentional use of task programming mechanisms and communication strategies in order to meet performance standards (Rico et al., 2018). Team coordination implies behaviors such as team members openly providing feedback to each other about the task environments and performance achievements, or communicating performance goal adjustment to meet unexpected situations (Rico et al., 2008; Jarzabkowski et al., 2012; Marques-Quinteiro et al., 2013).

Studies established the existence of a positive relationship between cohesion and team coordination (e.g., LePine et al., 2008). Cohesive teams have stronger social ties and experience less affective conflict, and the connectedness between team members facilitates team planning and information elaboration over time (Festinger, 1950). Thus, cohesion might be a coordination catalyst because it increases team members’ connectedness and facilitates their interaction and open communication, both of which are needed for coordination (Ensley et al., 2002).

According to the former rationales, cohesion will function as an initial condition for coordination. Evidence supporting this can be found in Zaccaro et al. (1995), who found that high task-cohesive teams invest more time in planning and information exchange during the planning period and communicate task-relevant information more frequently during the performance period than low task-cohesive teams did. These findings suggest that team coordination can be predicted over time by cohesion measured at the beginning of the performance cycle. Accordingly, we argue that at the beginning of a team performance cycle cohesion will function as an initial enabling condition promoting positive coordination trajectories over time. Thus, we hypothesize that:

*Hypothesis 3:* The level of team cohesion at the beginning of the performance cycle is positively related with the level of change in team coordination over time.

There are two major theories in the teamwork literature that allow us to theorize about the nature of team coordination development: Arrow et al.’s (2000) tCAS theory and Gersick (1991) punctuated equilibrium theory of team development. Both theories suggest that team coordination development is characterized by short periods of radical change happening halfway across the performance cycle, alternating with periods of stability where change is either smooth or nonexistent. This means that teams often spend the first half of a project or mission using a team coordination strategy and wait until halfway into that same project or mission to reformulate how they are sharing information and implementing decisions. Most interestingly, these dynamics should happen systematically, regardless of the duration of the teams’ performance cycles (e.g., minutes to months) or the number and length of meetings that the teams have at the beginning of the team performance cycle (Gersick, 1991).

Thus, we anticipate that the dynamics of team coordination over time are characterized by a discontinuity; that is, sudden, abrupt changes in coordination at the midpoint of the team performance cycle (Gersick, 1991; Arrow et al., 2000). Such discontinuity should happen because of the way teams develop and mature over time (Gersick, 1991; Arrow et al., 2000). Once a team is assembled, team members are likely to dedicate time learning how to work together, and how to relate with each other. During this period, team members will engage in team coordination behaviors, only making small adjustments until they finally reach self-organization, which is an orderly state that emerges almost spontaneously from the interactions between team members and often leads to higher performance (Kozlowski et al., 1999; Arrow et al., 2000). Whereas limited, there is empirical evidence revealing the occurrence of discontinuities in the way team processes change over time. In this regard, studies from the tCAS literature reported that team processes such as team learning (Rebelo et al., 2016) and team coordination (Guastello and Guastello, 1998) display discontinuous shifts. In addition, very recent research found that team action patterns (a proxy of team task coordination) exhibit discontinuous growth trajectories over time (Uitdewilligen et al., 2018).

Before self-organization is reached and when team members perceive the team has spent half of the time available to conclude a project or a mission, the team will go through a short period of disruptive change (Gersick, 1991). During this period, the quantity and quality of the feedback that is shared among team members increases. Team members learn from their own performance across the first half of the performance cycle and devise a new strategy to improve their performance in the second half. Through feedback and learning, team members develop a new shared understanding of the team and task reality, which should have direct influence on the quality of team coordination (e.g., Guastello and Guastello, 1998; Arrow et al., 2000). Once the team has self-organized by finding a new way of coordinating and performing, the team enters the second half of the team performance cycle and the number of modifications that team members do to their coordination strategy are more-or-less constant until the end of the team performance cycle (Gersick, 1991). Hence, building on these theories we hypothesize that coordination will display a smooth and incremental trajectory during the first and second half of the team performance cycle and that a discontinuity will take place at the midterm.

*Hypothesis 4:* The developmental dynamics of team coordination over time will display a discontinuous and linear trajectory, with a major change happening halfway across the performance cycle.

### Team Cohesion, Team Coordination, and Team Performance Over Time

We argued above that at the beginning of a team performance cycle, cohesion will function as an initial enabling condition promoting both coordination and performance trajectories over time. We expect that during the first half of the team performance cycle, team cohesion will be positively related with smooth and incremental changes in team coordination levels. Team cohesion gives teams the necessary plasticity to work through difficult situations without team member loss or process failures and facilitates coordination (Zaccaro et al., 1995; Kozlowski et al., 1999; Maynard et al., 2015). These changes should also be related with fluctuations in the level of team performance until halfway through the team performance cycle. However, as teams learn how to coordinate to perform their tasks, performance will vary because team members might not adopt the best coordination strategy from the beginning (Gersick, 1991; Guastello and Guastello, 1998). With the minimum entropy principle in consideration, fluctuations in team performance are likely for teams that perform high early in the team performance cycle (Guastello et al., 2013). The extent to which such nonlinear trajectories happen will be related with team cohesion as an initial condition.

At the midterm of the team performance cycle, teams tend to experience a radical increase in team coordination behaviors (Gersick, 1991). For teams who display a greater increase in the level of team coordination halfway through the team performance cycle and are capable of maintaining or slightly improving that level across the second half, team performance should preserve its nonlinear variability over time. Most importantly, cohesion will be beneficial to the evolution of coordination and team performance because the stronger connectedness between team members will ease the flow of valuable information within the team (Zaccaro et al., 1995; Arrow et al., 2000). Team members will elaborate more on what strategy they should follow to pursue teams’ goals and will coordinate wittingly in order to assure that the team is on the right track (Reagans and McEvily, 2003; Greer, 2012). For teams capable of effectively coordinating, it is expected that they will achieve higher performance over time (e.g., Arrow et al., 2000). We hypothesize that:

*Hypothesis 5:* The level of team cohesion at the beginning of the team performance cycle is positively related with the level of continuous and nonlinear change in team performance over time and this relationship is mediated by discontinuous and linear change in team coordination over time.

## Materials and Methods

### Research Context

Data collection took place during the first stage of a business simulation competition where each team had to run an entire company with the aim of achieving the highest investment performance. The criterion measured was the investment “return” for the original shareholders. On the first day of the competition, the market share value of every participating team was the same and the business market in which they competed was identical. Teams experienced real world-like events, such as currency devaluation, a hostile takeover or strikes.

Participants received all information necessary about the rules and the gaming environment 1 month before the competition began. Two weeks before the start of the competition, participating teams received two training sessions. This gave team members time to become familiar with the task and with each other. On day 1 of the competition teams received a general report that characterized their company and the business environment in which they were competing. During the competition, participants made top management decisions, analyzed financial and economic indicators, interacted with the different functional areas of a company (e.g., finance, human resource management, marketing), and were made aware of the impact their decisions had on the organization itself. During the competition, teams made 66 decisions weekly related to marketing, production, personnel, purchasing, and finance. Teams were also given a vast array of data to consider before making any decision. As in real financial markets, the competing companies’ stock trading was sensitive to the decisions made by the company’s management team. Teams had to upload their decisions to the competition online platform on the last day of the week, and received a report about their companies and their rivals’ performance 24 h later. The winner was the team that finished with the highest simulated share price. Teams were given absolute freedom to organize their work.

The business game competition where the participants of this study were enrolled is a high-fidelity simulation of a business company embedded in a virtual stock market abided by exactly the same rules of a real market. It offers an optimal data collection environment for the testing of new theory because experimenters have more control and data accessibility than in naturalistic settings (Marlow et al., 2017). In addition, the adoption of simulations has been proven highly effective in I/O Psychology and Human Factors research, and the number of empirical studies showing that simulations are most beneficial for research and training is growing (e.g., Uitdewilligen et al., 2018).

### Participants

A total of 158 teams comprised of 509 individuals participated voluntarily in this study (26% of the original population: 512 teams integrating 2163 individuals). Team size ranged between 3 (7.6%), 4 (28.5%), and 5 (63.4%) members (*M* = 4.56, *SD* = 0.64). The age of team members varied between 18 and 60 years old (*M* = 29.51, *SD* = 9.31), and 46% of the participants were women. Regarding experience in participating in previous editions of this business game competition, 69.4% of the participants had never been enrolled before, 17.8% had been enrolled once, and 12.6% had been enrolled in 3–10 editions. Regarding education, 53% of the participants had one college degree and 5.1% had at least two (Ph.D. = 0.4%, Master = 3.7, MBA = 1.0%). Fifty-four percent of the participants had (or were taking) a degree in a management-related program (15.7% of which were from General Management), and 26.1% of the participants had (or were taking) a degree in an engineering-related program. Finally, regarding team type, 51.3% of the teams were comprised of only professional workers coming from business companies, 44.8% were only integrated students (undergraduates and graduates), and 3.9% were mixed (i.e., professional workers and students).

### Design and Procedure

This study follows a longitudinal and correlational design because we collected data in more than three occasions over time (Roe, 2008), and we did not manipulate the independent variable (i.e., team cohesion). The business game competition lasted for five consecutive weeks. In light of Roe (2008) and Marks et al. (2001), the 5 weeks represented a full performance cycle, while each week represented one performance episode. Week 1 was the onset or beginning of the performance cycle, while week 5 was the end or offset of the performance cycle.

**FIGURE 1:**
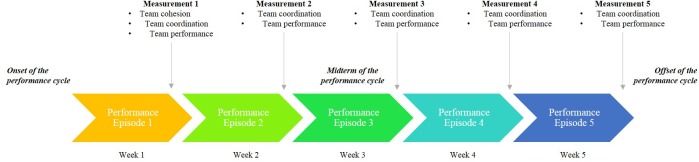
Illustration of the temporal structure of the business game competition and the data collection process.

We approached the designing of our study following methodological recommendations by Ployhart and Vandenberg (2010), and Ramos-Villagrasa et al. (2018) pointing to the need that longitudinal studies should be driven by (a) available theory informing which is the more adequate direction of causality between variables (e.g., Mathieu et al., 2015), or when certain forms of change are likelier to happen (e.g., Gersick, 1991), (b) the research question that is being pursuit (e.g., will team coordination dynamics mediate the relationship between initial team cohesion and team performance dynamics, from the beginning until the end of the performance cycle that is the business game competition?), (c) the nature of the variables under examination (e.g., psychological constructs and performance measures), and (d) practicality (e.g., when/how/for how long can data collection be performed). Because our research question was to study how team cohesion as an initial condition relates with change in team performance over time, through team coordination over time, we needed to ensure that (1) team cohesion was measured in the beginning of the business game competition (i.e., beginning of the team performance cycle), (2) team coordination and team performance were measured across the entire performance cycle (i.e., on each of the five performance episodes), and (3) that how and when each variable was collected reflected the causal relationship being hypothesized (i.e., team cohesion » team coordination » team performance). Whereas it could be argued that measuring team cohesion, team coordination, and team performance all together on week 1, and team coordination and team performance all together on weeks 2–5; could raise common method concerns and doubts about the assumption of causality, these were avoided (1) by measuring team cohesion in the first week of the business game competition, (2) by measuring team coordination and team performance in all 5 weeks, and (3) because team cohesion was measured first and team coordination was measured before teams could receive their weekly performance report (hence preventing that same-week team performance would input team coordination self-reports). Additionally, team cohesion and team coordination can be reliably measured through psychological scales such as the ones we have used. More, while the timing to measure team cohesion had to be at the end of the first performance episode (week 1) for practicality reasons (i.e., we could not measure it before), the timing to measure team coordination had to be at the end of each of the five performance episodes to allow us to know the teams’ overall coordination in each performance episode. The link to the online questionnaires remained active until participants received their performance report. Figure 1 illustrates the data collection process throughout the business game competition.

Finally, participants applied for the competition as intact teams coming from business companies and universities. This is why team familiarity was regarded as a control variable in our study. Participation in the competition was voluntary, and participants were invited to enroll upon registering for the event via email.

### Measures

Team members were asked to share their level of agreement regarding cohesion and coordination using a Likert-type scale ranging from *totally disagree* (1) to *totally agree* (7). Team cohesion as an initial condition, as well as team member familiarity and demographic variables, was measured in the first week (performance episode 1) of the business game competition. Team coordination was measured every week, from the beginning (performance episode 1) until the end (performance episode 5) of the business game competition. Team performance was objectively measured. As team coordination, it was measured on a weekly basis.

#### Team Cohesion

Team cohesion was measured as a multidimensional construct, using three items from the group environment questionnaire based on the saturation level of the items shown in Carless and DePaola (2000). One item measured *task cohesion* (“Our team is united in trying to reach its goals for performance in the competition),” one item measured *social cohesion* (“Our team likes to spend time together when we are not working”), and one item measured *individual attraction to the group* (“For me, this team is one of the most important social groups I belong to”). The three-items had acceptable reliability, α = 0.70. Since teams were formed 1 month before the start of the competition and had the opportunity to train together for the competition, they had enough time to establish cohesion (Festinger, 1950). Team cohesion as an initial condition was measured at the end of the first week of the competition.

#### Team Coordination

Team coordination was measured over 5 weeks using four items developed by West et al. (2004): “we are aware of what we want to accomplish,” “we debate the best ways to get things done,” “we meet several times to guarantee effective cooperation and communication,” and “we share task related information with each other.” The four-items had good reliability, α_week__1_ = 0.84, α_week__2_ = 0.81, α_week__3_ = 0.82, α_week__4_ = 0.82, and α_week__5_ = 0.84.

#### Team Performance

To win the competition teams had to manage the company in such a way that provided the highest investment performance at the end of the simulation. The investment performance reflects the return on investment to the respective investors, not only by stock market capitalization, but also after considering the issue or repurchase of shares and the dividends distributed. The measure of team performance was based on each team’s company stock share price at the end of the competition. This was automatically calculated by the computer program running the virtual environment in which teams competed.

#### Control Variables

Because participating teams could have a previous history of working together and past performance predicts future performance, team familiarity and initial team performance were controlled (LePine et al., 2008). Team performance was examined using the intercept of the team performance’s growth model. Team familiarity was measured with one item asking participants about the percentage of team members they already knew before enrolling. Responses could range from *I am not familiar with any of them* (0%) to *I am totally familiar with all of them* (100%).

### Aggregation

Before proceeding with data aggregation, we examined the within-group agreement index *r*_wg_ (James et al., 1984) and the intra-class correlation coefficients (ICC 1 and ICC 2; Bliese, 2000) to decide whether to proceed with data aggregation (Kozlowski and Klein, 2000).

### Analysis

#### Missing Data

In this study, the attrition level for individual responses varied between 31% (week 1) and 60% (week 5). The overall percentage of incomplete cases was 74.64%, and the overall percentage of incomplete values was 43.55%. The attrition level for team aggregated responses varied between 1% (week 1) and 15.2% (week 5). The overall percentage of incomplete cases was 19.05%, and the overall percentage of incomplete values was 2.34%. Decisions regarding how to handle missing data should be established by examining their pattern (Graham, 2009; Schlomer et al., 2010): missing completely at random (MCAR), missing at random (MAR), and not MAR (NMAR). Thus, to determine the pattern of missing data, we performed the Little (1988) MCAR test using the missing values analysis command option in SPSS 22. We obtained a non-significant chi-square value for $\chi_{\mathrm{individual responses}}^{2}$ = 599.601, *df* = 651, *p* = 0.926, and for $\chi_{\mathrm{team responses}}^{2}$ = 45.894, *df* = 38, *p* = 0.178, indicating that the pattern of missing data is MCAR (Little, 1988). MCAR is considered as a nonproblematic missing data pattern that is best managed by using sophisticated stochastic imputation methods such as full information maximum likelihood (FIML) (Graham, 2009; Ployhart and Vandenberg, 2010; Muthén and Muthén, 2012).

#### Assessing Configural Invariance

We performed a confirmatory factor analysis (CFA) for each team process measured at each time point, separately. The factorial structure was determined based on the theoretical operationalization of team explicit coordination by Rico et al. (2008) and West et al. (2004). The goodness-of-fit was estimated using the Chi-square index (χ^2^), which evaluates the magnitude of discrepancy between the sample and fitted covariance matrices. To complement the use of the Chi-square index, three additional model fit indexes were considered: the root mean square approximated error (RMSEA), which measures the discrepancy between the hypothesized model and data by degrees of freedom (values ≤ 0.08 suggest goodness of fit, although some authors have argued that values ≤ 0.06 are ideal); the comparative fit index (CFI), which carries out the comparison between the fit of the hypothesized model and that of a basic model being represented by a null model (it can range between 0.90 and 1.00, with ideal fit values being ≥ 0.95); and the standardized root mean square of residual (SRMR), that should be ≤0.08 for good fit (Hu and Bentler, 1998).

Table 2 shows the model fit for team coordination over 5 weeks. Hu and Bentler (1998) suggest that decisions about the adequacy of model fit should be done using a minimum 2-index strategy to reject reasonable proportions of various types of true-population and misspecified models. The results of the CFA for team coordination show RMSEA values ≤0.17, which are above the minimum cutoff criteria point to assume good model fit. Nevertheless, Hu and Bentler (1998) suggest that the RMSEA alone is less preferable when dealing with very small sample sizes ≤600 and that combining the CFI and the SRMR can provide a more reliable alternative. The results displayed in Table 2 suggest that for all cases except one (team coordination in the second week, CFI = 0.90), both CFI and SRMR index values were within the recommended cutoff criteria point to assume good model fit (Hu and Bentler, 1998). Therefore, we considered that the factorial structure for each team coordination measurement, for every week, had an acceptable model fit. Having established configural invariance, we then tested measurement invariance (Chen, 2007).

#### Assessing Measurement Invariance

We followed a four-step approach in which four models were tested for team coordination (Chan, 1998; Lance et al., 2000; Muthén and Muthén, 2012; van de Schoot et al., 2012): Model 1, where only the factor loadings were set as equal over time but the intercepts were allowed to differ between weeks; Model 2, where only the intercepts were equal over time, but the factor loadings were allowed to differ between weeks. Model 3, where the loadings and intercepts were constrained to be equal over time; and Model 4, where the residual variances were also fixed to be equal over time [for further detail please regard, Lance et al. (2000) and van de Schoot et al. (2012)]. The minimum fit requirements to assume measurement invariance are that the fit of Model 3 cannot be significantly worse than Model 1 or Model 2 (Lance et al., 2000).

Since the χ^2^ difference test is very sensitive to sample size, the testing of measurement invariance should be done with alternative fit indexes such as RMSEA, CFI, and SRMR. Following Chen (2007), for sample sizes ≤600, measurement invariance of factor loadings (e.g., Model 1) can be assumed when one observes a change of ≤0.010 in CFI, supplemented by a change of ≤0.015 in RMSEA, or a change of ≤0.030 in SRMR; and measurement invariance of intercept (e.g., Model 3) or residual (e.g., Model 4) invariance can be assumed when one observes a change of ≤0.010 in CFI, supplemented by a change of ≤0.015 in RMSEA, or a change of ≤0.010 in SRMR. Among the three indexes, CFI should be regarded as the main criterion to determine measurement invariance because RMSEA and SRMR tend to over reject invariant models (Chen, 2007). Given the small sample size, bootstrap estimation with 5000 cases was used. The results in Table 2 suggest that both ΔCFI and ΔRMSEA for team coordination were null, or equal to 0.01. This is close to optimal fit conditions since both indexes did not change regardless of accumulating model constraints (Chen, 2007). Therefore, measurement invariance for team coordination was assumed.

Before we proceed to the main results section, it is important to highlight that performing the measurement invariance tests is computationally demanding and benefits from large sample sizes (*N* > 1000). Therefore, weak model fit under measurement invariance testing should not be considered as a model rejection criterion, especially when performed with small samples. Indeed, despite the weak model fit displayed in Table 2 for the measurement invariance test, what should be regarded is the stability of the model fit indicators across models. As suggested by Hopwood and Donnellan (2010), strict rejections of models based upon rigid adherence to fit index cutoffs should be considered only with regard to theoretical or substantive issues. Since the model fit for configural invariance was adequate, and keeping in mind that the testing of measurement invariance was performed using a small sample size (Hu and Bentler, 1998; Chen, 2007), we decided to proceed with further analyses.

## Results

Table 1 displays the main descriptive statistics, correlations, and reliability scores for all variables studied. The results suggest that 29 out of 66 correlations were positive and significant, *r*s ≥ 0.20, *p*s ≤ 0.01, and team cohesion was negatively and significantly correlated with team performance on week 2, *r* = −0.02, *p* < 0.05.

**Table 1:** Unstandardized correlations for team cohesion, team coordination, and team performance.

	1	2	3	4	5	6	7	8	9	10	11	*M*	*SD*
Team familiarity	1	–	–	–	–	–	–	–	–	–	–	74.29	25.62
Team cohesion	0.33^∗∗^	1	–	–	–	–	–	–	–	–	–	5.26	0.84
Team coordination time 1	0.07	0.56^∗∗^	1	–	–	–	–	–	–	–	–	5.71	0.70
Team coordination time 2	0.04	0.29^∗∗^	0.55^∗∗^	1	–	–	–	–	–	–	–	5.80	0.69
Team coordination time 3	−0.09	0.25^∗∗^	0.48^∗∗^	0.48^∗∗^	1	–	–	–	–	–	–	5.57	0.82
Team coordination time 4	−0.010	0.21^∗^	0.37^∗∗^	0.58^∗∗^	0.65^∗∗^	1	–	–	–	–	–	5.76	0.72
Team coordination time 5	0.04	14	0.34^∗∗^	0.46^∗∗^	0.51^∗∗^	0.71^∗∗^	1	–		–	–	5.65	0.84
Team performance time 1	0.12	−0.02	0.06	0.12	−0.05	0.06	−0.03	1	–	–	–	4.47	2.29
Team performance time 2	0.12	−0.02^∗^	0.00	0.09	0.07	0.07	0.04	0.46^∗∗^	1	–	–	4.56	2.23
Team performance time 3	0.17^∗^	−0.15	0.00	−0.02	0.07	0.20^∗^	0.18	0.36^∗∗^	0.72^∗∗^	1	–	4.73	2.27
Team performance time 4	0.18^∗^	−0.13	0.02	−0.01	0.07	0.14	0.14	0.26^∗∗^	0.65^∗∗^	0.86^∗∗^	1	4.85	2.23
Team performance time 5	0.12	−0.13	0.04	0.01	0.07	0.17	0.20^∗^	0.21^∗∗^	0.59^∗∗^	0.79^∗∗^	0.91^∗∗^	4.96	2.22

*N_teams_ = 158; ^∗^p < 0.05, ^∗∗^p < 0.01.*

Table 3 displays the aggregation indexes for team cohesion and team coordination. The results show that both the *r*_wg_ index and the ICC (1) index were according to standards (James et al., 1984; Bliese, 2000), hence suggesting that the aggregation of data was possible. Regarding the values of the ICC (2) index, these were below the recommended threshold of 0.70, which can be explained by the small sample size of the teams examined in our research. Bliese (2000) argues that small ICC (2) values are not an impediment to data aggregation. For constructs with low ICC (2), the strength of the relationship between research variables might be attenuated. Thus, low ICC (2) values may have made the testing of team level relationships somewhat conservative.

**Table 2:** Configural invariance and measurement invariance for team coordination.

	Week	χ^2^(*df*)	RMSEA	CFI	SRMR
Configural invariance	1	11.58 (2)^∗^	0.12	0.97	0.03
	2	20.12 (2)^∗^	0.17	0.90	0.05
	3	11.84 (2)^∗^	0.13	0.93	0.04
	4	7.36 (2)^∗^	0.11	0.97	0.03
	5	2.21 (2)	0.02	0.99	0.01
Measurement invariance	Model 1	862.58 (181)^∗^	0.10	0.79	0.09
	Model 2	888.83 (181)^∗^	0.10	0.79	0.10
	Model 3	940.27 (188)^∗^	0.10	0.77	0.10
	Model 4	956.10 (192)^∗^	0.10	0.77	0.11

*N_individuals_ = 509; ^∗^p < 0.001. For measurement invariance testing, bootstrap estimation with 5000 cases was used. Model 1: only factor loadings constrained to be equal over time; Model 2: only intercepts constrained to be equal over time; Model 3: both factor loadings and intercepts constrained to be equal over time; and Model 4: factor loadings, intercepts, and residual variances constrained to be equal over time.*

**Table 3:** Aggregation indexes for team cohesion and team coordination.

	*r*_wg_, ICC(1), ICC(2)				
	Time 1	Time 2	Time 3	Time 4	Time 5
Cohesion	0.82, 0.24, 0.59	–	–	–	–
Coordination	0.83, 0.14, 0.44	0.88, 0.25, 0.60	0.83, 0.12, 0.39	0.85, 0.11, 0.37	0.88, 0.22, 0.55

*N_individuals_ = 509.*

### The Dynamics of Team Processes

To determine the dynamics of change for team coordination and team performance we built four competing models describing different forms of change: linear change (Model 1), quadratic change (Model 2), nonlinear change (Model 3), and discontinuous change (Model 4). The linear and quadratic temporal terms were modeled using polynomials. This means that whereas the linear trend was modeled by defining each temporal term as 0, 1, 2, 3, and 4 (with 0 marking the intercept or initial status of the research variable), the quadratic term was modeled by squaring the linear time metric, i.e., 0, 1, 4, 9, 16. Additionally, to model nonlinearity we fixed the onset and offset temporal terms of each team process as 0 and 1, allowing all other terms to adopt nonlinear trajectories (in case there were any). To model discontinuity, because this was hypothesized to occur between the third and fourth week of the competition, we modeled change as 0, 0, 0, 1, 1. This allows us to determine if there is a discontinuity (either positive or negative) in the slope of team coordination on the third week of the business game competition (for in-depth description of these approaches, please regard Ployhart and Vandenberg, 2010).

Table 4 summarizes the modeling procedure for each of the four growth models and reports the growth model fit statistics for each of them. The results suggested that team coordination, χ^2^ (*df*) = 21.98 (10), *p* = 0.015, RMSEA = 0.09, CFI = 0.93, SRMR = 0.09, was best described by a continuous linear change model (Model 1); and team performance was best described by a continuous nonlinear change model (Model 3), χ^2^ (*df*) = 19.62 (7), *p* < 0.01, RMSEA = 0.11, CFI = 0.97, SRMR = 0.07. These findings do not support hypothesis 4 and support hypothesis 2. The model fit for team coordination and team performance was good because at least two model fit indexes scored within recommended cutoff point criteria (Hu and Bentler, 1998). Although the RMSEA was above the recommended threshold of 0.08, it can still be considered a fair model fit (Hu and Bentler, 1998), especially because RMSEA is very sensitive to small sample sizes. Based on these results, the linear continuous model for team coordination and the nonlinear continuous model for team performance were set as the baseline growth models in following analyses (Lance et al., 2000).

**Table 4:** Model fit for the dynamics of growth trajectories of team coordination and team performance.

Variable	Nature of change	Form of change	Modeling of change	χ^2^ (*df*)	RMSEA	CFI	SRMR
Team coordination	Continuous	Linear	0,1,2,3,4	21.98 (10), *p* = 0.015	0.09	0.93	0.09
		Quadratic	0,1,4,9,16	16.63 (6), *p* = 0.012	0.11	0.94	0.09
		Nonlinear	0 – – – 1	23.82 (7), *p* = 0.001	0.12	0.90	0.10
	Discontinuous	Linear	0,0,0,1,1	36.31 (10), *p* < 0.001	0.13	0.84	0.15
Team performance	Continuous	Linear	0,1,2,3,4	46.27 (10), *p* < 0.001	0.15	0.91	0.15
		Quadratic	0,1,4,9,16	3.94 (6), *p* = 0.685	0.00	1.00	0.03
		Nonlinear	0 – – – 1	19.62 (7), *p* < 0.001	0.11	0.97	0.07
	Discontinuous	Linear	0,0,0,1,1	77.75 (10), *p* < 0.001	0.21	0.83	0.22

*N_teams_ = 158. The – in the nonlinear modeling, between 0 and 1, represents the freely estimated parameters in the model.*

#### The Descriptives of Change

The latent growth model parameter estimates (i.e., factor means, variances, and covariances) were regarded with the goal of further characterizing the nature of growth trajectories for team coordination and team performance (Lance et al., 2000). The results displayed in Table 5 show that the mean, μs = −0.03, *SE* = 0.05, *p* < 0.001, 95% CI [5.651; 5.824], and the variance, σ = 0.30, *SE* = 0.05, *p* < 0.001, 95% CI [0.218; 0.372], of the intercept for team coordination were statistically significant. Similarly, the results also suggest that the mean, μ = 4.42, *SE* = 0.18, *p* < 0.01, 95% CI [4.129; 4.714], and the variance, σ = 3.36, *SE* = 0.54, *p* < 0.01, 95% CI [2.478; 4.250], of the intercept for team performance were statistically significant. Thus, there were interteam and intrateam differences in team coordination and team performance at the beginning of the performance cycle.

**Table 5:** Unstandardized simple growth parameter estimates and model fit.

	Team coordination				Team performance			
	Estimate	*p*	*SE*	95% CI	Estimate	*p*	*SE*	95% CI
Intercept μ	−0.03	^∗∗^	0.05	5.651; 5.824	4.42	^∗^	0.18	4.129; 4.714
Intercept σ	0.30	^∗∗^	0.05	0.218; 0.372	3.36	^∗^	0.54	0.074; 0.791
Slope μ	−0.02	0.30	0.02	−0.049; 0.011	0.43	0.047	0.22	2.478; 4.250
Slope σ	0.03	^∗∗^	0.01	0.018; 0.040	5.05	^∗^	1.02	3.371; 6.726
Cov μ	−0.03	0.08	0.02	−0.051; −0.001	−1.87	^∗∗^	0.18	−2.949; −0.782
Model fit	χ^2^ (*df*)	RMSEA	CFI	SRMR	χ^2^ (*df*)	RMSEA	CFI	SRMR
	21.98 (10)^∗^	0.09	0.93	0.09	16.62 (7)^∗∗^	0.11	0.97	0.07

*N_teams_ = 158. ^∗∗^p < 0.001, ^∗^p < 0.01. μ regards mean (e.g., intercept mean; slope mean). σ regards variance (e.g., intercept variance; slope variance).*

The analysis of the descriptives of change shows that whereas the slope factor mean for team coordination was not significant, μ = −0.02, *SE* = 0.02, *p* = 0.30, 95% CI (−0.049; 0.011), the slope factor mean for team performance was positive and significant, μ = 0.43, *SE* = 0.22, *p* = 0.05, 95% CI (0.073; 0.791). Furthermore, the slope factor variances for team coordination and team performance were also positive and significant, σs ≥ 0.03, *SE*s ≥ 0.01, *p*s ≤ 0.01, 95% CI (≥3.371; ≤6.726). This result suggests that team coordination between teams did not change significantly over time, but that team performance did. Additionally, team coordination and team performance positively and significantly changed within teams; meaning that some teams significantly improved both their coordination and performance over time.

Finally, the results of the simple latent growth curve models for team coordination and team performance over time suggest that both constructs had a negative and significant covariance between the intercept and the slope, cov_coordination_ = −0.03, *SE*s = 0.02, *p* = 0.08, 95% CI (−0.051; −0.001); cov_performance_ = −1.87, *SE*s = 0.18, *p* < 0.001, 95% CI (2.949; −0.782). The analysis of change descriptives reveals that the higher the level of team coordination and team performance at the beginning of the team performance cycle, the less they coordinated and performed well over time.

**FIGURE 2:**
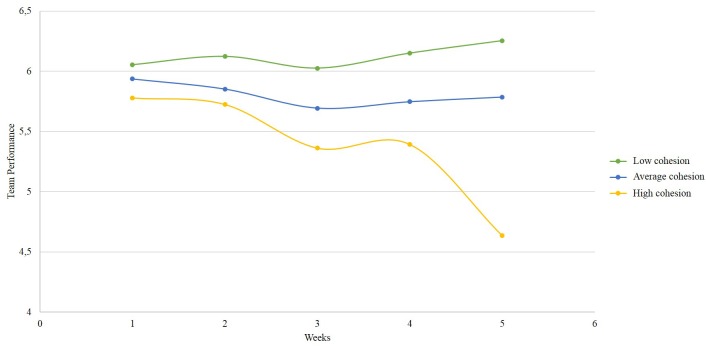
Interteam growth trajectories for team performance over time, when initial team cohesion is low, medium, and high.

Figure 2, 3 summarize how team cohesion as an initial condition (i.e., low, average, and high) relates with different trajectories for team performance and team coordination over time. Figure 4 summarizes the temporal mediation results.

**FIGURE 3:**
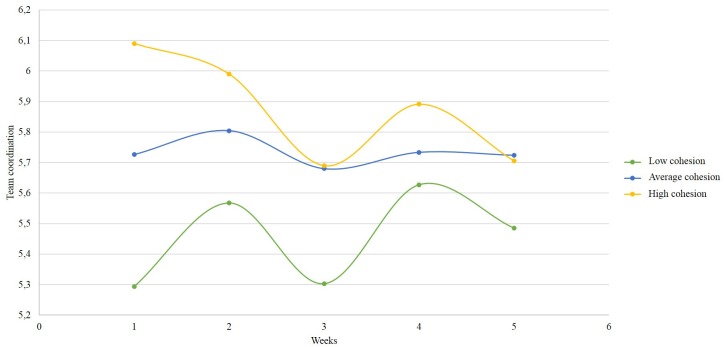
Interteam growth trajectories for team coordination over time, when initial team cohesion is low, medium, and high.

**FIGURE 4:**
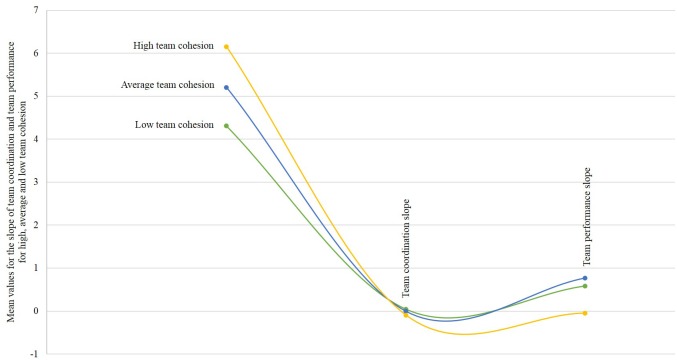
Interteam mediation growth trajectories for the relationship between team cohesion and team performance, through team coordination, when initial team cohesion is low, medium, and high.

To summarize, whereas team cohesion is an initial condition to teamwork dynamics, our findings contradict the initial hypothesis that cohesion should enable teamwork and suggest that an excess of team cohesion at the beginning of a performance cycle may impair the way team coordination and team performance change over time.

#### Team Cohesion as an Initial Condition

Mediation latent growth curve models (MLGCMs) are particularly useful to test for mediations where individual trajectories (i.e., trajectories between teams) of change over time are described, and where intra-individual change (i.e., trajectories within teams) is expected (von Soest and Hagtvet, 2011). As in simpler mediation models, mediation in MLGCM is supported when the variable *X* changes the level of the mediator *M*, and the change in the mediator influences the level of the outcome variable *Y* over time. The mediational process can be modeled as the effect of *X* influencing the growth of *Y*, indirectly through the growth of *M* (Cheong et al., 2003; Selig and Preacher, 2009; von Soest and Hagtvet, 2011). Following von Soest and Hagtvet (2011), growth curves (i.e., slopes/trajectories) and the MLGCM were built based on unstandardized mean scores from team cohesion (*X*), team coordination (*M*), and team performance (*Y*). To deal with missing data we used a FIML estimator (Muthén and Muthén, 2012). Bootstrapping was used to estimate all bias-corrected CIs based on 5000 bootstrap samples (von Soest and Hagtvet, 2011). Likewise, bias-corrected bootstrap CIs were computed for mediation effects. For this purpose, we combined in Mplus the “model indirect” and the “cinterval” commands (von Soest and Hagtvet, 2011).

The overall model fit for the mediation model was satisfactory, χ^2^ (53) = 119.23, *p* < 0.001, RMSEA = 0.09, CFI = 0.93, SRMR = 0.09. The results displayed in Table 5 suggest that team cohesion was negatively related with change in team coordination over time, *B* = −0.07, *SE* = 0.02, *p* < 0.001, 95% CI (−0.102; −0.037), and unrelated with change in team performance over time, *B* = −0.18, *SE* = 0.14, *p* = 0.194, 95% CI (−0.503; 0.000). These findings do not support hypotheses 1 and 3. The results also suggest that change in team coordination over time is positively related with change in team performance over time, *B* = 3.22, *SE* = 1.08, *p* = 0.001, 95% CI (1.385; 4.962). Finally, the research findings reported in Table 6 suggest that change in team coordination over time negatively and significantly mediates the relationship between team cohesion and change in team performance over time, *B* = −0.23, *SE* = 0.10, *p* = 0.02, 95% CI (−0.455; −0.115). This finding does not support hypotheses 5.

**Table 6:** Unstandardized mediation latent growth curve modeling (hypotheses testing).

	*B*	*SE*	*p*	95% CI
Team cohesion regressed on the slope of team coordination.	−0.07	0.02	0.001	−0.102; −0.037
Slope of team coordination regressed on the slope of team performance.	3.22	0.98	<0.001	1.385; 4.962
Team cohesion regressed on the slope of team performance.	−0.18	0.29	0.835	−0.503; 0.000
Indirect effect for the slope of team coordination.	−0.23	0.10	0.022	−0.455; −0.115

*N_teams_ = 158. Mediation model was tested controlling for the intercept of team performance, B = −0.13, SE = 0.09, p = 0.145, 95% CI [−0.281; 0.009], and team familiarity, B = 0.02, SE = 0.01, p < 0.001, 95% CI [0.012; 0.029], on the slope of team performance.*

## Discussion

The aim of this study was to examine how team cohesion contributes to performance trajectories over time, through coordination trajectories. More specifically, we tested whether coordination longitudinally mediates the relationship between cohesion and performance in a sample of teams enrolled in a business simulation competition. Overall, we found that cohesion is negatively related with team coordination and team performance over time. These findings suggest that higher cohesiveness might work as a disabling condition to coordination and performance trajectories in business teams. Although it was not part of our initial theorizing, finding that the level of team coordination and team performance at the beginning of the team performance cycle is negatively related with the level of change in both constructs over time further highlights that the extent to which team members engage in coordination behaviors such as sharing information or having meetings, or perform very highly at the beginning of a team performance cycle, can also be initial *disabling* conditions to the teamwork phenomena over time. These unexpected results have important theoretical and practical implications that deserve consideration.

### Theoretical Implications

Although our findings diverge from previous research suggesting a positive relationship between team cohesion and team performance, they are not contradictory but rather complementary. For instance, in Mathieu et al. (2015) the relationship between cohesion and performance was regarded longitudinally in the sense that the authors focused on the co-evolution of both constructs over time. Their findings suggest that cohesion and performance co-evolve positively over time, and their temporal relationship works better when cohesion is an antecedent of performance. Additionally, in Mathieu et al. (2015) the mean values for cohesion and performance at the beginning and end of the business simulation suggest that low cohesion management teams (sample 2) were achieving higher performance. Although this issue was not addressed by the authors, such findings are consistent with our results regarding the relationship between the level of cohesion at the beginning of a performance cycle, and the evolution of performance over time. It is possible that while looking at cohesion and performance as co-evolving constructs a positive relationship is found; when cohesion is regarded as an initial condition to the evolution of performance over time a negative relationship is found instead. This interpretation aligns with longitudinal theory suggesting that depending on how researchers study the temporal dynamics of their variables of interest, the relationship between the two same constructs may yield different patterns of results (Roe, 2008; Cronin et al., 2011; Kozlowski, 2015; Navarro et al., 2015).

We find additional explanations of our results in extant literature. Accordingly, Wise (2014) reported an inverse curvilinear relationship between team cohesion and team performance, in which team performance is lower at high and low levels of team cohesion and optimal at average levels of team cohesion. Research by Gargiulo and Benassi (2000) also suggests that highly cohesive communication networks are less likely to adapt their coordination strategies to situational requirements, thus performing poorly compared to moderately cohesive communication networks.

Another explanation of our pattern of findings could be that the high levels of team cohesion (*M* = 5.26, *SD* = 0.84) reported by participating teams in this study might have functioned as a heuristic for team members to determine to what extent the team was coordinating and performing well. In this line, Artinger et al. (2015) suggest that heuristics play a fundamental role in driving adaptive decision-making in managerial work environments. The authors advocate that heuristics provide a simple, less cognitively loaded, source of information from which fast decisions can be reached. However, such decisions can result in either a positive or negative outcome. This argument finds support in research by Callaway and Esser (1984) and Mullen et al. (1994) who found that more cohesive groups often render poorer decision-making outcomes. Thus, such findings align with *t*CAS theory (Arrow et al., 2000) and teamwork development theorization proposing that teams performing in complex work environments (such as it is the case of *our* teams enrolled in the business game competition) perform high when the ties between team members are strong enough to keep them working together, but not too strong to prevent them to openly question and debate their ideas or be proactive in looking for external resources that might stimulate team performance (Kozlowski et al., 1999).

Our results also have implications for the study of team coordination. As previously stated, coordination is dependent on team members’ ability to communicate openly, share relevant information, and plan (Ensley et al., 2002; Rico et al., 2008). However, the inefficiencies of high cohesion that cause a decrease in coordination capacity can harm team performance as well, given that team members will be less capable of articulating key information and task direct efforts (Esser, 1998). For teams whose initial cohesion levels are high, it might well be that biasing group phenomena such as groupthink and polarization interfere with the quality of the decisions that determine performance. Indeed, highly cohesive teams might avoid task/cognitive conflict because they believe that conflict will hamper team processes and outcomes. Rather than openly communicating, constructively confronting and exchanging ideas during performance episodes, team members will stick to the plan and avoid any kind of confrontation that threatens the team. Such passivity could be another good candidate in explaining why high initial levels of cohesion cause a reduction in task coordination and performance over time. Hardy et al. (2005) examined the relationship between cohesion, processes, and performance in sports teams; they found that 56% of the participants explicitly reported that cohesion was detrimental for both individual and collective dynamics. Participants reported that too much social cohesion caused wasted time during training, goal-related problems, and team member social isolation (e.g., ugly duckling effect; scapegoat effect). And importantly, participants also reported that high task cohesion often caused decreased member contribution to the team or task, reduced social relations, and communication inefficiencies.

Particularly, communication inefficiencies have been shown to be detrimental to coordination over time and to performance as well (e.g., Gargiulo and Benassi, 2000). Thus, when team members fail to assess relevant information, it is likely that errors will occur while communicating and planning (e.g., Grote et al., 2010). Such errors also result in a collective inability to build accurate team situational models, which results in poor performance (Stout et al., 1999; Rico et al., 2008). The increase of communication inefficiencies also brings several problems to task coordination because the decrease in team members’ collective awareness reduces the likelihood that team members will attend task inputs and fellow team members needs in a timely manner (Driskell and Salas, 1992).

To summarize, most studies on cohesion and cohesion sub-dimensions have found empirical support for the benefits of cohesion. These results have been received without much questioning, probably because the idea of cohesion as a good thing is intuitively appealing and apparently logical. Although our findings suggest that too much cohesion is bad for team functioning, we cannot say that cohesion is not functional for coordination and performance. In fact, we show how cohesion is certainly important, but only to a certain extent. Accordingly, as elaborated above our findings echo previous research showing evidence of cohesion as having a negative effect on teamwork dynamics (e.g., Mullen et al., 1994; Wise, 2014). One important detail in our findings that cannot go unnoticed is that while a cross-sectional examination of the relationship between initial cohesion and coordination showed a positive relationship between both constructs (Table 1), using a longitudinal approach allowed us to identify a negative relationship. The evolution of coordination and performance over time worsened for teams whose levels of initial cohesion were higher. These findings raise an interesting point; they suggest that the way theory is built on the relationship between cohesion and teamwork dynamics should be firmly rooted in longitudinal data (Cronin et al., 2011; Kozlowski and Chao, 2012). Furthermore, these findings suggest that the way relationships between constructs are theorized and examined is heavily dependent on how levels of analysis and time are considered (Roe, 2008; Navarro et al., 2015).

### Practical Implications

Looking at our results and how they build on existing practitioner literature, a key implication of this research is that for those planning to assemble a new project team or start a business venture, assuring an average level rather than a maximum level of team cohesion at the beginning of their task will pay off for key team processes and team performance over time.

Another implication is that this study may increase HR managers and team leaders’ awareness that using cross-sectional versus longitudinal lenses to examine cohesion might result in conflicting information about the way teamwork dynamics will change across a full performance episode. Indeed, practitioners should note that managing performance over time requires the use of longitudinal data analysis in order to gain a more reliable perception of what is occurring.

Our findings also suggest that measuring cohesion at the beginning of a project might help toward designing better training and coaching support programs. Our results suggest that training coordination skills on teams is a valuable and important human resources management practice because being able to effectively coordinate over time is a baseline condition to achieve higher team performance in the workplace (Rico et al., 2018).

### Limitations and Future Research

As in every empirical study, this research is not without its limitations. The first limitation of this research regards the fact that the unique features of the research context (i.e., a simulation) suggest caution when generalizing the research findings to real business organizations, and other work environments. Indeed, while the simulation emulates many of the characteristics of real business environments (e.g., the decisions that teams make about the way they manage their company will affect the company’s value in the stock market), there are no real-world consequences resulting from good or bad managerial decisions (e.g., the company going bankrupt and employees losing their jobs). However, the adoption of high-fidelity simulations like the business game competition in which our data were collected is not new to the study of teamwork phenomena such as team cohesion, team coordination, or team performance (e.g., Zaccaro et al., 1995; Mathieu et al., 2015). More, there is considerable growth in the number of empirical studies showing that high-fidelity simulations are most beneficial for learning and training because participants behave as if they were performing in *real life* (Marlow et al., 2017). This is particularly true for those simulations that best recreate the real-life contexts in which participants will have to perform. The closeness between simulation and reality increases the simulation’s ecological validity, meaning that the likelihood that participants will behave in a similar way to how they would behave when performing in real environments is very high (Leemkuil and De Jong, 2012). Additionally, although we could not find any empirical papers addressing the extent to which the results of high-fidelity business simulations replicate in real business organizations, we found one study by Lievens and Patterson (2011), where the authors suggest that high-fidelity simulations are powerful predictors of job candidates’ future job performance. This suggests that how individuals behave and perform during high-fidelity simulations can be replicated in real jobs.

Another limitation in our study could be that our sample is partially formed by teams of undergraduate students which also may affect the generalizability of our findings (Peterson, 2001). However, some teams in our sample were also entirely (or partially) composed of professional workers. In many organizations, work teams might have different degrees of maturation or professional experience. It is likely that some teams have very little experience (e.g., recently graduated team members), while others are composed of senior individuals that are highly experienced (Kozlowski et al., 1999). As in the previous limitation, we believe that this study replicates real-world conditions by considering teams that have highly experienced (professional workers) and poorly experienced (undergraduate students) teams. Therefore, we think that the fact our sample included students is not a serious threat to the generalizability of our findings. Besides, Druckman and Kam (2011) have systematically compared differences in research findings, between studies using students versus non-students as participants, thus finding little to none significant differences between them^1^.

A third limitation in this study is missing data. Missing data often raises several concerns regarding how reliable research findings can be; because the results might be contingent on the characteristics of the individuals that decide to participate in the study rather than the real relationship the constructs have (Graham, 2009). However, the fact that our missing data pattern was MCAR and given the utilization of a FIML estimation to test our hypotheses, the chance that missing data had an effect on the research outcomes is very small (Graham, 2009).

Having found no support for most of our research hypotheses might hinder perceptions about the potential contribution of this study. However, recent work by authors such as Franco et al. (2014) have raised a warning regarding the potentially biasing effect of avoiding the publication of research findings that support the null hypothesis, especially in the social sciences. They stress the negative biasing effects that such practice has in knowledge development because it limits our full understanding of social systems. Thus, the communication and dissemination of unexpected or contradictory findings are important to improve social sciences (Scargle, 1999).

Finally, we see three research opportunities that are worth exploring since they could help solving most of the aforementioned limitations. To test the robustness and generalizability of our research findings, future studies could examine what will happen if: (a) individuals are randomly assigned to teams, (b) individual characteristics such as task expertise are considered, and (c) data are collected in real business environments. All of these could be addressed with two studies. Study 1 could focus on (a) and (b), while Study 2 could focus on (c). Both (a) and (b) could be addressed in an experimental setting where the main task would be performing the same business game competition that we use, and where team member allocation (random vs. intact) and expertise (low expertise vs. high expertise) are regarded as independent variables. For instance, it could be that for teams whose team members are less familiar with each other, high expertise will be fundamental to ensure more positive team coordination and team performance trajectories across the performance cycle (e.g., Zaccaro et al., 1995; Mathieu et al., 2015). More, building on recent work by Maynard et al. (2019), by measuring team cohesion (task and social) as a covariate, researchers could also learn how both team familiarity and team cohesion contribute to teamwork processes such as team coordination and team performance. Once Study 1 is performed, Study 2 could be conducted with the goal of replicating and extending our findings using a quasi-experimental setting where newly assembled teams are compared with teams with a long existence.

Besides these suggestions, we also encourage researchers to explore (a) how each sub-dimension of cohesion influences the evolution of coordination and performance over time, and (b) what would be the temporal dynamics of team cohesion, team coordination, and team performance if an event that triggered adaptation would happen at the halfway point transition of team performance cycle (Maynard et al., 2015). Social cohesion is the sub-dimension that mostly relates to the quality of the relationships within the team (Greer, 2012). Hence, it is likely that initial social cohesion will have a stronger detrimental effect on task coordination and performance over time, than task cohesion will. In our study, we could not know the extent to which participants worked together every week, and how many hours they spent together on social activities. Future studies could have access to this information and regard it as proxies of team cohesion. How each cohesion dimension contributes to coordination and performance trajectories over time might also depend on the team development stage (Kozlowski et al., 1999), and even the extent to which the need for team adaptation is triggered halfway through the team performance cycle (Maynard et al., 2015). For less experienced teams with little familiarity among team members, social cohesion and interpersonal attraction might be the most important dimensions of cohesion that need to be leveraged. The sooner team members establish stronger social ties, the better they will be able to engage in collaborative learning and performance. Engaging in such behaviors will then facilitate the development of team mental models, which are needed for task coordination and performance. Over time, as teams gain experience and forge stronger interpersonal connections, task cohesion might emerge as a more relevant dimension of team cohesion. This is because it will give team members a sense of agreement and stability that will reduce stress and cognitive load and give team members the opportunity to focus on task or goal-directed behaviors. Still, if a dramatic shift occurs halfway through the team performance cycle, high social cohesion might be fundamental to prevent team coordination breakdowns and severe performance losses (Maynard et al., 2015).

## Conclusion

Understanding the dynamics characterizing teamwork and team members’ interrelations requires considering the role of time and the incorporation of initial conditions triggering team processes trajectories (Arrow et al., 2000; Hackman, 2012; Ramos-Villagrasa et al., 2018). This study contributes to the teamwork literature by showing that the more cohesive a team is, the greater the likelihood that the team will see its ability to coordinate and perform impaired over time.

## Ethics Statement

This study was carried out in accordance with the recommendations of ethical guidelines of the Ethical Committee (CE) at ISCTE Instituto Universitário, with written informed consent from all subjects. All subjects gave written informed consent in accordance with the Declaration of Helsinki.

## Author Contributions

All authors were involved in the righting of the theoretical background and discussion sections. PM-Q was also responsible for analyzing and reporting the results.

## Conflict of Interest Statement

The authors declare that the research was conducted in the absence of any commercial or financial relationships that could be construed as a potential conflict of interest.

## Footnotes

1 The results of the independent samples *t*-test for team cohesion suggest that professional teams and student teams did not differ on this regard, *t* (151) = −1.14, *p =* 0.312. The results of the two-way repeated measures ANOVA suggest that, although that team coordination, *F*(4,151) = 14.07, *p* < 0.001, and team performance, *F*(4,151) = 2.42, *p =* 0.048, changed over time, change was not qualified by an interaction between time and group type for team coordination, *F*(4,151) = 0.67, *p =* 0.617, and team performance, *F*(4,151) = 0.48, *p* = 0.753.
